# Development and validation of a risk score (Delay-7) to predict the occurrence of a treatment delay following cycle 1 chemotherapy

**DOI:** 10.1016/j.esmoop.2022.100743

**Published:** 2022-12-19

**Authors:** P. Chambers, M.D. Forster, A. Patel, N. Duncan, E. Kipps, I.C.K. Wong, Y. Jani, L. Wei

**Affiliations:** 1 Department of Practice and Policy, School of Pharmacy, University College London, London, UK; 2 The Centre for Medicines Optimisation Research and Education, University College London NHS Foundation Trust, London, UK; 3 University College London Cancer Institute, London, UK; 4 The Royal Marsden Hospital, London, UK; 5 University Hospitals Birmingham NHS Foundation Trust, Birmingham, UK

**Keywords:** chemotherapy, dose-delay, toxicity, breast cancer, colorectal cancer, diffuse-large B-cell lymphoma

## Abstract

**Background:**

The risk of toxicity-related dose delays, with cancer treatment, should be included as part of pretreatment education and be considered by clinicians upon prescribing chemotherapy. An objective measure of individual risk could influence clinical decisions, such as escalation of standard supportive care and stratification of some patients, to receive proactive toxicity monitoring.

**Patients and methods:**

We developed a logistic regression prediction model (Delay-7) to assess the overall risk of a chemotherapy dose delay of 7 days for patients receiving first-line treatments for breast, colorectal and diffuse large B-cell lymphoma. Delay-7 included hospital treated, age at the start of chemotherapy, gender, ethnicity, body mass index, cancer diagnosis, chemotherapy regimen, colony stimulating factor use, first cycle dose modifications and baseline blood values. Baseline blood values included neutrophils, platelets, haemoglobin, creatinine and bilirubin. Shrinkage was used to adjust for overoptimism of predictor effects. For internal validation (of the full models in the development data) we computed the ability of the models to discriminate between those with and without poor outcomes (*c*-statistic), and the agreement between predicted and observed risk (calibration slope). Net benefit was used to understand the risk thresholds where the model would perform better than the ‘treat all’ or ‘treat none’ strategies.

**Results:**

A total of 4604 patients were included in our study of whom 628 (13.6%) incurred a 7-day delay to the second cycle of chemotherapy. Delay-7 showed good discrimination and calibration, with *c*-statistic of 0.68 (95% confidence interval 0.66-0.7), following internal validation and calibration-in-the-large of −0.006.

**Conclusions:**

Delay-7 predicts a patient’s individualised risk of a treatment-related delay at cycle two of treatment. The score can be used to stratify interventions to reduce the occurrence of treatment-related toxicity.

## Highlights

• Delays to systemic anticancer treatment commonly occur, reducing overall dose intensity.
• Individualised physiological factors can be used to predict.
• A risk score can bring together factors and accurately predict the risk of treatment delays.
• The score should be validated and future use would be to individualise toxicity management for patients.

## Introduction

Systemic anticancer treatments (SACT) can cause haematological and non-haematological toxicity; where the latter can occur in 70% of patients treated.^1^ Severe toxicity is undesirable as it will result in delays to subsequent treatments, thereby reducing patient experience and increasing health care costs.^2^ The occurrence of these delays, in the curative setting, may also result in suboptimal therapy, with emerging evidence—for some cancers—of the importance of accurate treatment timing.^3^^,^^4^

The risk of dose delays with cancer treatment should be included as part of pretreatment education and be considered by clinicians upon prescribing, as this information can influence decisions such as escalation of standard supportive care or improved adherence by patients. Personalised approaches to toxicity management would therefore be supported.

Early detection and management of toxicities is a strategy that has been researched and demonstrated success at reducing the incidence of dose delays.^2^^,^^3^^,^^5^ Various methods exist including nurse-led monitoring or utilisation of electronic patient-reported outcome measures (PROMS). These strategies involve resource in implementation and may not be of benefit to all patients, but if patients were accurately identified, these interventions could reduce acute care use, morbidity and costs.

Although several risk scores are available to predict specific toxic effects such as febrile neutropenia, none have, to date, investigated dose delays as a whole.^6^, ^7^, ^8^, ^9^, ^10^ Additionally, most studies have been conducted only at single institutions^6^^,^^9^ and one population study was found on acute admissions in the palliative setting; but to date there is no score available for patients receiving treatment of potentially curable cancers where an early intervention could influence their overall time on treatment.^8^

In this study, we developed and validated the prediction of chemotherapy dose delays (Delay-7) to estimate the probability of delay occurrence.

## Methods

### Creation of cohort

Our data was derived from the electronic prescribing (EP) systems from four academic hospitals in England. In total, these hospitals treat around 18 000 cancer chemotherapy patients per year. Data were extracted for outcome measures and candidate predictors, identified from a systematic review and through consultation with expert clinicians and patients.

### Eligibility criteria

Data were included for patients aged ≥18, identified through the chemotherapy EP system at each hospital for the period of 1 January 2013 to 1 January 2018. The first chemotherapy treatment date from the EP system was used as the index date for entry to the cohort during the study period. The study data were restricted to the following three tumour groups: breast, colorectal and diffuse large B-cell lymphoma, identified using the International Classification of Diseases 10th Revision (ICD-10) coding of C50, C83, C19, C19, C20 and C21. Justification for this was that this would be a large population receiving relatively standard treatments, enabling us to develop a risk model. In the case of breast cancer, we only included those with early breast cancer (stages 1-3), and in the case of colorectal cancers, we included all patients receiving their first treatments for any stage disease. Although the colorectal population included some metastatic patients, disease control and response rate are believed to be optimised in this group through achievement of a dose intensity of >80%.^11^ For all tumour groups, only patients receiving first-line treatments were included. We restricted our inclusion to the following treatments: epirubicin and cyclophosphamide (EC) plus or minus fluorouracil (FEC); docetaxel plus or minus cyclophosphamide (TC, T-FEC); irinotecan modified de Gramont (IrMdG); oxaliplatin modified de Gramont (FOLFOX) and combinations including irinotecan; oxaliplatin and capecitabine (OXCAP); rituximab, cyclophosphamide, vincristine and prednisolone (R-CHOP). Data were excluded for patients where only one treatment cycle was administered. Additionally, we excluded patients where the second cycle had been administered over 60 days from the index date as this type of delay was outside the scope of this research.

### Ethics and data use

Heath Research Authority (HRA) approvals were required and granted on 24 November 2017 (IRAS 226078). The study was registered with European Network of Centres for Pharmacoepidemiology and Pharmacovigilance (ENCePP) study number EUPAS35413.

### Outcome

The study outcome of interest was an administration delay for 7 days for treatment cycle 2. A delay of 7 days was considered as a suitable period that was used by clinicians for toxicity-related delays.^12^ Expert clinicians identified that delays under 7 days could be an effect of poor scheduling. The outcome was generated through comparing the number of days between the first and second cycle with the intended cycle length of the treatment prescribed.

### Predictive variables

The predictors to toxicity-related delays^13^ were hospital treated, age at the start of chemotherapy, gender, ethnicity, body mass index (BMI), presence of cardiovascular comorbidity or diabetes, performance status, cancer group, chemotherapy regimen and associated cycle length, colony stimulating factor (CSF) use, first cycle dose modifications and baseline blood values. Baseline blood values were for neutrophils, platelets, haemoglobin, creatinine and bilirubin.

All laboratory values, BMI and age were in a continuous format. Where possible, continuous predictors were not categorised. Where this was the case linearity was tested to meet the assumptions of logistic regression and plots enabled the identification of outliers. Assessments of plausibility were made where outliers were present; an outlier was defined as any value that was 1.5 times more than the third quartile or lower than the first quartile. Any erroneous outliers were considered as missing.

To balance the statistical and clinical robustness of the model we decided to categorise continuous variables for laboratory values. Categorisation is generally not recommended^14^ as it results in loss of information on predictor effects particularly when two categories are used (dichotomisation). We justified categorisation firstly, however, as the categories used are well recognised and firmly established in clinical practice.^15^ Secondly, in routine practice there are more than two grading categories used, meaning less loss of information in contrast to two categories. Lastly, strong evidence exists that low neutrophils and haemoglobin, or high bilirubin or creatinine are associated with dose delays, meaning truncation of outliers was inappropriate.

### Sample size

Sample size of a prognostic model development study is informed by three factors: anticipated prevalence of the outcome (treatment delays), desired sensitivity of the model to the outcome and the precision of the 95% confidence interval around the sensitivity of the model.^16^

To maximise statistical power, we used all patient data from the four hospitals that met our inclusion criteria in model development. To reduce overfitting of the model, the number of variables for inclusion in model development were restricted to 10 events per variable. Dose delays in these tumour groups included were understood to occur in 10%-15% of the cancer population. In total, 28 predictors were included in our model and therefore a dataset including 280 events was required equating to a minimum sample size of 2800 patients (assuming a 10% rate). We included a total of 4604 patients in the analysis.

### Missing data

Our cohort had missing information on vascular comorbidity, BMI, neutrophils, platelets, bilirubin and creatinine. Some 50% of the data for vascular comorbidity were missing and on assessment it was found that these originated from one hospital, meaning it was inappropriate to impute this data. The hospital was a large academic centre treating only patients with cancer, and missingness was associated with data imputation policies, where the recording of comorbidities was not mandated. Missing data for other variables were believed to be missing at random and equated to <10%. We used multiple imputation to replace missing values by using a chained equation approach based on all candidate predictors excluding vascular comorbidity. We created 10 imputed datasets for missing variables that were then combined across all datasets by using Rubin’s rule to obtain final model estimates.^17^

### Statistical analysis for model development and validation

We treated occurrence of a dose delay as a binary outcome measure. For each of the candidate predictors, we used a univariable logistic regression model to calculate the unadjusted odds ratio. To derive our risk prediction model, we included all candidate predictors in a multivariable logistic regression model.

We assessed the performance of the model in terms of the *c*-statistic and calibration slope (where 1.00 is ideal). The *c*-statistic represents the probability that for any randomly selected pair of patients with and without a dose delay, the patient who had a dose delay had a higher predicted risk. A value of 0.50 represents no discrimination and 1.00 represents perfect discrimination. This process was repeated in our imputed and complete case dataset and a sensitivity analysis was undertaken comparing the coefficients obtained.

To validate our developed model and correct measures of predictive performance for optimism (overfitting) we used bootstrapping, using 200 samples of the derivation data. We then repeated the model development process in each bootstrap sample. To account for overfitting during the development process, we multiplied the original β coefficients by the uniform shrinkage factor in the final model. At this point we re-estimated the intercept based on the shrunken β coefficients to ensure that overall calibration was maintained, producing a final model.

Lastly, we carried out a net benefit analysis (which was not prespecified), to evaluate the potential clinical value of using Delay-7 to inform decision making. This analysis assumes that the threshold probability of the occurrence of dose delay at which a patient or clinician would opt for intervention is informative in terms of false positives and false negatives. This is then used to calculate the net benefit of the model across a wide range of threshold probabilities. The most basic interpretation of a decision curve is that the model with the highest net benefit at a particular threshold has the highest clinical value. In our study, the decision curve analysis assessed the potential clinical benefits of using the Delay-7 model to select patients for alternative models of care. In this analysis three scenarios are compared: selecting all patients for the intervention (treat all), selecting no patients (treat none) and selecting patients using the predictive model. The *x*-axis depicts the threshold probability, which is chosen by the decision maker. The *y*-axis depicts the net benefit of each strategy, which is expressed in terms of the value of true positives.^18^

We used Stata version 15 (*Stata Statistical Software: Release 15*. College Station, TX: StataCorp LLC) for all statistical analyses. This study was conducted and reported in line with the Transparent Reporting of a multivariate prediction model for Individual Prediction or Diagnosis (TRIPOD) guidelines.^14^

## Results

### Study population

In our cohort from hospitals located in England, we analysed information on 4604 patients after excluding 447 patients where a second cycle of treatment was not recorded to be administered (see Supplementary Table S1, available at https://doi.org/10.1016/j.esmoop.2022.100743). Of the 4604 patients, there were 628 (13.6%) occurrences of 7-day delays. Table 1 summarises the characteristics of the study population. Women represented 69% of the cohort, due to the inclusion of breast cancer.

**Table 1:** Characteristics of patients in the development cohort and univariable associations between each variable and the outcome of treatment delay following cycle one of treatment

Predictor	No delay at cycle 2 *N* = 3976	Delay to cycle 2 *N* = 628	Odds ratio	95% CI	*P* value
Hospital, *n* (%)					
1	1450 (36.4)	314 (50)	Ref	Ref	Ref
2	1106 (27.8)	179 (28.5)	0.75	0.61-0.91	0.004
3	867 (22)	91 (14.5)	0.48	0.38-0.62	<0.0005
4	553 (14)	44 (7)	0.37	0.26-0.50	<0.0005
Cancer group, *n* (%)					
Breast	1837 (46.2)	185 (29.5)	Ref	Ref	Ref
Colorectal	1517 (38.2)	387 (61.6)	1.10	0.82-1.52	0.48
DLBCL	622 (15.6)	56 (8.9)	2.80	2.10-3.80	<0.0005
Chemotherapy, *n* (%)					
FEC	1134 (28.5)	110 (17.5)	Ref	Ref	Ref
EC	465 (11.7)	55 (8.8)	0.82	0.58-1.50	0.26
FOLFOXIRI	13 (0.3)	8 (1.3)	0.71	0.42-1.21	0.21
IrMdG	479 (12)	152 (24.2)	5.20	2.07-13.1	<0.0005
OXCAP	354 (8.9)	64 (10.2)	2.70	1.92-3.75	<0.0005
FOLFOX	671 (16.9)	163 (26)	1.53	1.04-2.25	0.031
R-CHOP	622 (15.6)	56 (8.9)	2.05	1.48-2.85	<0.0005
Docetaxel	238 (6)	20 (3.2)	0.76	0.51-1.13	0.17
Cycle length, *n* (%)					
14 Days	1238 (31.1)	346 (55.1)	Ref	Ref	Ref
21 Days	2738 (68.9)	282 (44.9)	0.37	0.31-0.44	<0.0005
First cycle dose modification, *n* (%)					
No	3958 (99.5)	623 (99.2)	Ref	Ref	Ref
Yes	18 (0.5)	5 (0.8)	1.76	0.65-4.7	0.3
Use of CSF, *n* (%)					
Yes	1175 (30.5)	126 (19.2)	Ref	Ref	Ref
No	2801 (69.5)	502 (81)	0.60	0.49-0.74	<0.0005
Age in years at start of chemotherapy (skewed)	Median 55 Range (18-90)	Median 59 Range (19-88)	1.01	1-1.02	<0.0005
Gender, *n* (%)					
Female	2736 (68.8)	387 (61.6)	Ref	Ref	Ref
Male	1240 (31.2)	241 (38.4)	1.37	1.15-1.63	<0.0005
Body mass index	Mean 27, SD 5.9	Mean 26, SD 5.8	0.99	0.98-1.01	0.49
Ethnicity, *n* (%)					
aAny non-white	847 (21.3)	107 (17)	Ref	Ref	Ref
White (any)	3129 (78.7)	521 (83)	1.32	1.05-1.64	0.14
Neutrophil count (×10^9^/l), *n* (%)					
<2	161(4.1)	30 (4.8)	Ref	Ref	Ref
2-7	2996 (75.3)	433 (69)	0.78	0.5-1.16	0.21
>7	811 (20.4)	165 (26.2)	1.09	0.7-1.67	0.7
Missing	8 (0.2)	0	—	—	—
Haemoglobin (g/dl), *n* (%)					
<8	23 (0.6)	3 (0.5)	Ref	Ref	Ref
8-10	284 (7.1)	60 (9.6)	1.62	0.47-5.6	0.44
>10	3576 (90)	556 (88.5)	1.2	0.35-4	0.78
Missing	93 (22.3)	9 (1.4)	—	—	—
Creatinine (μmol/l) , *n* (%)					
<110	3584 (90.1)	538 (85.7)	Ref	Ref	Ref
110-165	221 (5.6)	50 (8)	1.51	1.09-2.08	0.012
>165	115 (2.9)	32 (5.1)	1.85	1.24-2.80	0.003
Missing	56 (1.4)	8 (1.3)	—	—	—
Bilirubin (μmol/l), *n* (%)					
<22	3830 (96.3)	596 (95)	Ref	Ref	Ref
22-33	64 (1.6)	15 (2.4)	1.51	0.85-2.70	0.2
>33	37 (0.9)	6 (1)	1.04	0.44-2.50	0.09
Missing	45 (1.1)	11 (1.8)	—	—	—
Diabetes or cardiovascular comorbidity, *n* (%)					
No	2047 (61.5)	240 (38.2)			
Yes	479 (12.1)	74 (11.8)	Ref	Ref	
Missing	1450 (36.5)	314 (50)	1.30	0.99-1.74	0.053
Performance status, *n* (%)					
0	3538 (89)	532 (84.7)	Ref	Ref	Ref
1	368 (9.3)	77 (12.3)	1.40	1.07-1.81	0.013
2+	70 (1.44)	19 (3)	1.80	1.07-3.00	0.025

CI, confidence interval; CSF, colony stimulating factors; DLBCL, diffuse large B-cell lymphoma; EC, epirubicin and cyclophosphamide; FEC, fluorouracil, epirubicin, cyclophosphamide; FOLFOX, oxaliplatin and fluorouracil; FOLFOXIRI, fluorouracil, oxaliplatin, irinotecan; IrMdG, irinotecan modified de gramont: irinotecan, fluorouracil; OXCAP, oxaliplatin and capecitabine; R-CHOP, rituximab, cyclophosphamide, vincristine and prednisolone; SD, standard deviation.

a Other ethnicity included Asian, black, Chinese, mixed, other or unknown).

**Figure 1:**
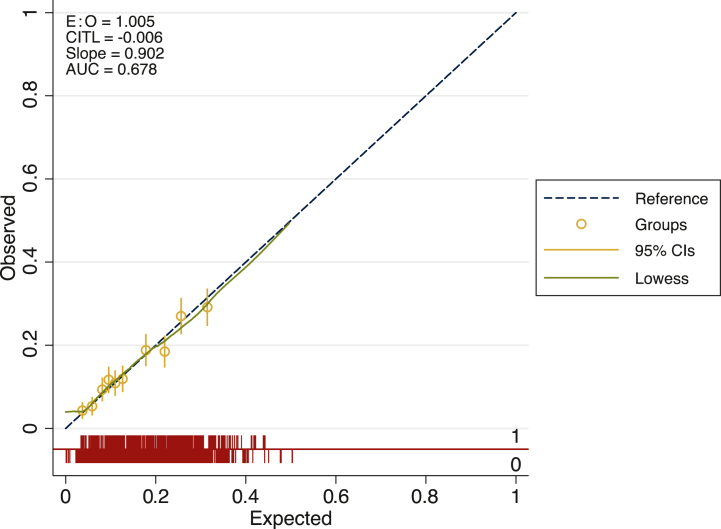
**Calibration plot for Delay-7.** **Notes:Following bootstrapping calibration remains strong and discrimination is similar.** AUC, area under the curve (or *c*-statistic); CI, confidence interval; CITL, calibration in the large; E : O, estimated to observed ratio.

Univariable associations between delays to treatment and potential predictors are also displayed in Table 1. Of the 44 candidate predictors (from 16 risk factors), 16 were statistically significantly associated with delays. FOLFOX and IrMdG, used widely in colorectal cancer (including advanced disease), showed significant associations with delays but with wide confidence intervals that could be attributed to the mixed population in this disease group. Table 2 shows the apparent and internal validation performance statistics of our risk prediction model developed using multivariable methods. After adjustment for optimism, our final risk prediction model was able to discriminate patients who were likely to encounter a delay with a *c*-statistic of 0.68 (95% confidence interval 0.66-0.70). The agreement between the observed and predicted proportion of events showed excellent apparent calibration following bootstrapping (Figure 1).

**Table 2:** Delay-7 model coefficients for the complete case cohort and imputed cohort

	Complete case			Imputed data		
Variable	Beta coefficient Complete case	SE Complete case	*P* value	Beta coefficient	SE	*P* value
Hospital						
1	Ref	Ref	Ref	Ref	Ref	Ref
2	−11.12	542.7	0.98	11.66	696.15	0.98
3	−11.64	542.7	0.98	12.15	696.15	0.98
4	−11.8	542.7	0.98	12.25	696.15	0.98
Chemotherapy						
EC	Ref	Ref	Ref	Ref	Ref	Ref
FEC	0.09	0.23	0.68	0.14	0.20	0.50
Docetaxel	−0.23	0.30	0.25	−0.10	0.30	0.70
FOLFOXIRI	1.57	0.69	0.02	1.84	0.60	0.03
IrMdG	0.62	0.45	0.17	0.60	0.38	0.11
OXCAP	0.43	0.30	0.15	0.50	0.27	0.26
FOLFOX	0.46	0.46	0.31	0.60	0.39	0.26
R-CHOP	−0.04	0.27	0.87	0.20	0.25	0.94
Cycle length						
14 Days	Ref	Ref	Ref	Ref	Ref	Ref
21 Days	−0.04	0.37	0.25	−0.48	0.30	0.12
Dose reduction received	−11	542	1.00	−11.5	696	1.00
CSF received	0.05	0.17	0.80	0.003	0.16	0.90
Body mass index	0.05	0.08	0.50	0.02	0.07	0.07
Age	0.01	0.04	0.22	0.003	0.10	0.10
Sex						
Female	Ref	Ref	Ref	Ref	Ref	Ref
Male	−0.23	0.12	0.06	−0.22	1.10	0.05
Ethnicity						
Non-white	Ref	Ref	Ref	Ref	Ref	Ref
White origin	0.14	0.12	0.26	0.14	0.12	0.27
Performance status						
0	Ref	Ref	Ref	Ref	Ref	Ref
1	0.16	0.15	0.28	0.12	0.14	0.30
2+	0.59	0.29	0.04	0.46	0.27	0.09
Neutrophils (×10^9^/l)						
<2	Ref	Ref	Ref	Ref	Ref	Ref
2-7	−0.61	0.23	0.01	−0.53	0.21	0.4
>7	−0.31	0.24	0.20	−0.29	0.23	0.1
Haemoglobin (g/dl)						
<8	Ref	Ref	Ref	Ref	Ref	Ref
8-10	0.18	0.65	0.79	0.12	0.67	0.86
>10	−0.01	0.64	1.00	0.04	0.66	1.00
Creatinine (μmol/l)						
<110	Ref	Ref	Ref	Ref	Ref	Ref
110-165	0.20	0.19	0.30	0.62	0.17	0.72
>165	0.18	0.24	0.46	0.16	0.22	0.46
Bilirubin (μmol/l)						
<22	Ref	Ref	Ref	Ref	Ref	Ref
22-33	0.40	0.34	0.24	0.36	0.30	0.24
>33	−0.06	0.47	0.90	0.96	0.46	0.84

CSF, colony stimulating factors; EC, epirubicin and cyclophosphamide; FEC, fluorouracil, epirubicin, cyclophosphamide; FOLFOX, oxaliplatin and fluorouracil; FOLFOXIRI, fluorouracil, oxaliplatin, irinotecan; IrMdG, irinotecan modified de gramont: irinotecan, fluorouracil; OXCAP, oxaliplatin and capecitabine; R-CHOP, rituximab, cyclophosphamide, vincristine and prednisolone; SE, standard error.

Table 3 presents the final model coefficients and Table 2 shows coefficients for each variable included in the final model for both the complete case and imputed datasets. The coefficients indicate the weighting that each factor has on the outcome. Notably, use of CSF showed little additive effect in this model although demonstrating a significant univariable association.

**Table 3:** Model performance statistics

	Test used	Delay-7 model	Following bootstrapping
Overall performance	Brier score	0.16	0.12
Discrimination	*c*-statistic	0.67 (0.65-0.71)	0.68 (0.66-0.70)
Calibration	Calibration in the large	0	—

**Figure 2:**
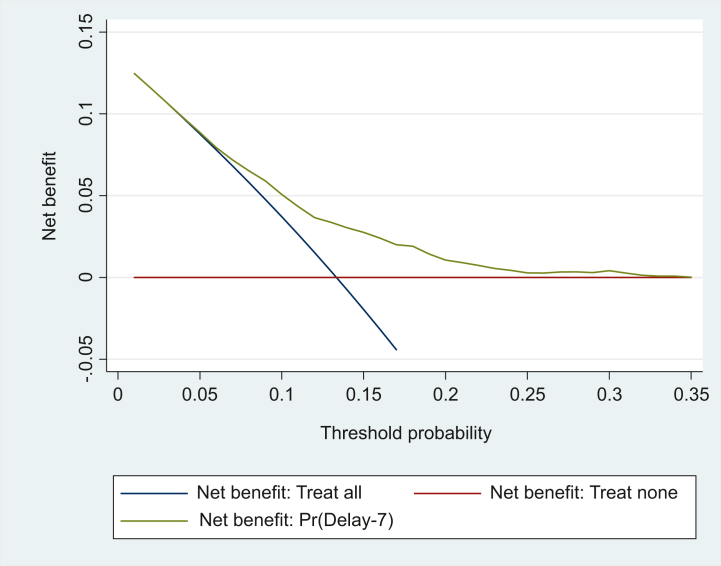
Decision curve for threshold probabilities.

Decision curve analysis for the Delay-7 score in the cohort is displayed in Figure 2. This decision curve demonstrates that selecting patients for an intervention using the Delay-7 had an appreciable net benefit compared with the treat all and treat none strategies for threshold probabilities that are <25%.

## Discussion

In this study, we developed and internally validated a score (Delay-7) to predict 7-day dose delays for patients receiving cancer chemotherapy. This is the first risk score that has been developed in the non-palliative setting and could support patients to receive timely treatments in future. The score was developed using a national representative dataset containing 4604 patients that was collected for this purpose and 13.6% of them experienced an event of dose delay. The included predictors in the score would be readily available to clinicians within any EP system and therefore could be calculated upon initiating treatment. We believe that Delay-7 can be used to improve both the informed consent process and help stratify patients to interventions that have demonstrated success at reducing the rates and severity of adverse events such as proactive monitoring and early interventions.

Proactive monitoring, either through nursing support or electronically, has demonstrated success in a number of studies, but these interventions require resource.^5^ Through using a stratification approach to identify patients, these interventions become more feasible. We believe that Delay-7 once validated could support the implementation of evidence-based interventions to improve the safety of patients receiving treatments.

Our work is important to future policy as the numbers of cancer patients increase year upon year.^19^ Using a model to direct interventions to those likely to have the occurrence of a delay due to toxicity would be both resourceful and improve safety and patient experience. A reduction in the occurrence of treatment delays for some patients may also improve their response to treatment,^4^ negating the need for future treatments. The decision to develop a generic model was through the understanding of the processes in the UK where toxicity advice and monitoring is led by nursing and pharmacy teams during treatment.^20^ Currently, the model is only applicable to the treatments researched in specific cancers and research around validation and implementation is planned. To be implemented in clinical practice, we acknowledge that there may be clinical hesitancy without the inclusion of tumour-specific attributes. Furthermore, the approach of developing a more cancer specific model will make validation studies more achievable through utilisation of national datasets.^8^ Our model requires a validation cohort to test that our score continues to perform in heterogenous populations. Future work will therefore explore international collaborations as other researchers have achieved^21^ to validate our work.

The discrimination of our score is similar to those published to predict febrile neutropenia^10^ and hospitalisations within 30 days of palliative treatment.^9^ Discriminatory ability is improved through the inclusion of predictive variables, and as the decision to delay treatment because of toxicity can be subjective, the inclusion of behavioural elements may have improved discriminatory ability.

The strengths of Delay-7 compared were size and methodological rigour, adhering to prespecified published protocols and reporting guidelines. We used age as a continuous variable whereas other models^10^^,^^22^ in other settings have dichotomised age (>65 years), limiting their transferability. We had a large number of events (*n* = 628) and this is reflected in the narrow confidence intervals retrieved from our performance statistics. Our study has some limitations. As we extracted data from individual hospitals, we could not ascertain any reasons for discontinuation and therefore could not include this as an endpoint in addition to delayed dose. Reasons that patients might not receive more than one cycle of treatment could be movement to a different hospital or choice to cease treatment rather than discontinuation due to toxicity. The high volume of missing data for comorbidity meant we not include this in our final score. Aforementioned, this could be addressed in a validation cohort through working with more comprehensive national datasets, examining the performance of a model that included and excluded comorbidity, utilising more alternative measures of comorbidity, based on availability.^23^ Most patients in our cohort were of a white ethnicity, meaning that confidence intervals around risk estimates for women of other individual ethnic groups were large. We therefore collapsed ethnicity groupings into white and not white in our model. In future work, we would like to further refine risk estimates for women of different ethnicities. Finally, through our investigations of variables we uncovered differences in hospitals and their rates of delays, meaning that simple alignment to both operational and clinical procedures such as appropriate threshold setting for haematological toxicity could result in reduced delays.^24^ We believe that through a combination of alignment of policies and tailoring toxicity support through use of our model, we will improve the relative dose intensity of treatments received.

### Conclusions

Delay-7 predicts the risk of delays of 7 days that can be used as a proxy measure for toxicity-related delays after the initiation of systemic therapy for cancer. The score quantifies an important risk of systemic therapy, which can improve the informed consent process. Validation of Delay-7 is required; however, once achieved the score could support accurate stratification of patients to preventative interventions.
