# Supplementary material for: Development and validation of a risk score (Delay-7) to predict the occurrence of a treatment delay following cycle 1 chemotherapy
Source: ESMO Open. 2022 Dec 19;8(1):100743. doi: 10.1016/j.esmoop.2022.100743 (PMC10024092; doi:10.1016/j.esmoop.2022.100743)
Supplement: Supplementary Table S1 [file mmc1.docx]

Supplementary table 1. Showing cycles recorded at each hospital.

| **Hospital** |  | **Cycles recorded** | | | | | **Total Patients** |
| --- | --- | --- | --- | --- | --- | --- | --- |
|  | **1** | **2** | **3** | **4** | **5** | **6 or more** |  |
| 1 | 326  (16%) | 321 (15%) | 540  (26%) | 145  (7%) | 102  (5%) | 656  (31%) | 2,090 |
| 2 | 41  (3%) | 51  (4%) | 78  (6%) | 119  (9%) | 99  (7%) | 938  (71%) | 1,326 |
| 3 | 50  (5%) | 50  (5%) | 87  (9%) | 88  (9%) | 35  (3%) | 697  (69%) | 1,008 |
| 4 | 30  (5%) | 25  (4%) | 56  (9%) | 53  (8%) | 15  (2%) | 450  (72%) | 627 |
